# Using prediction markets to forecast research evaluations

**DOI:** 10.1098/rsos.150287

**Published:** 2015-10-28

**Authors:** Marcus R. Munafo, Thomas Pfeiffer, Adam Altmejd, Emma Heikensten, Johan Almenberg, Alexander Bird, Yiling Chen, Brad Wilson, Magnus Johannesson, Anna Dreber

**Affiliations:** 1 MRC Integrative Epidemiology Unit at the University of Bristol, Bristol, UK; 2 UK Centre for Tobacco and Alcohol Studies, School of Experimental Psychology, University of Bristol, Bristol, UK; 3 New Zealand Institute for Advanced Study, Massey University, Palmerston North, New Zealand; 4 Wissenschaftskolleg zu Berlin, Berlin, Germany; 5 Department of Economics, Stockholm School of Economics, Stockholm, Sweden; 6 Sveriges Riksbank, Stockholm, Sweden; 7 Department of Philosophy, University of Bristol, Bristol, UK; 8 School of Engineering and Applied Sciences, Harvard University, Cambridge, MA, USA; 9 Consensus Point, Nashville, TN, USA

**Keywords:** research evaluation, prediction market, research excellence framework

## Abstract

The 2014 Research Excellence Framework (REF2014) was conducted to assess the quality of research carried out at higher education institutions in the UK over a 6 year period. However, the process was criticized for being expensive and bureaucratic, and it was argued that similar information could be obtained more simply from various existing metrics. We were interested in whether a prediction market on the outcome of REF2014 for 33 chemistry departments in the UK would provide information similar to that obtained during the REF2014 process. Prediction markets have become increasingly popular as a means of capturing what is colloquially known as the ‘wisdom of crowds’, and enable individuals to trade ‘bets’ on whether a specific outcome will occur or not. These have been shown to be successful at predicting various outcomes in a number of domains (e.g. sport, entertainment and politics), but have rarely been tested against outcomes based on expert judgements such as those that formed the basis of REF2014.

## 1. Background

The 2014 Research Excellence Framework (REF2014) was intended to assess the quality of research carried out at higher education institutions (HEIs) in the UK over a 6 year period. The stated aims of REF2014 included to: produce robust indicators of research excellence, provide a basis for distributing funding, reduce the administrative burden on institutions compared with previous exercises, and avoid creating undesirable behavioural incentives. Submissions were graded as 4* (world-leading), 3* (internationally excellent), 2* (recognized internationally), 1* (recognized nationally) and unclassified (falls below the standard of nationally recognized work). Institutions submitted returns across a number of Units of Assessments (UoAs), such as chemistry. Each submission comprised, roughly: a number of individual researchers (who each had to submit four outputs, such as publications), a number of ‘impact’ case studies (defined as examples of the impact that academic research has had outside of academia, with the number of case studies required depending on the number of researchers returned), and a statement about the department’s research environment (including data on research income, research students, etc.).

During the period prior to the REF2014 submission date of 31st October 2013, concerns grew that the process was failing with respect to at least some of its stated objectives. In particular, there was evidence that HEIs were attempting to ‘game’ the system, for example, by recruiting academics with established research records who would enhance a submission to a particular UoA, and employing large numbers of staff on the minimum part-time contract that qualified them for submission to REF2014 [1]. The process was also criticized for being expensive and bureaucratic—some HEIs, for example, hired teams of science writers to prepare impact case studies, while academics spent considerable time engaged in ‘mock’ REF exercises in an attempt to maximize the strength of their submission. Other concerns included the extent to which four outputs could fully capture the breadth and depth of academic research output, and how impact could be measured. One critical concern was whether the exercise would reveal much beyond what was largely already known by the academic community regarding the relative standing of different institutions and departments. This last point is particularly important given the costs associated with large-scale evaluation exercises such as REF2014, including indirect costs such as the time commitment of experts required to review submissions and make complex qualitative judgements on these.

Prediction markets have become increasingly popular as a means of capturing what is colloquially known as the ‘wisdom of crowds’ in a number of domains, including sport, entertainment and politics, where they have shown to perform well in predicting outcomes [2–4]. A prediction market enables individuals to trade ‘bets’ on whether a specific outcome will occur or not. If many participants are trading in such a market, market prices will generate a prediction of the outcome, based on the aggregated information of the participants. While prediction markets have been discussed as a potential tool that could be used in science [5–7], relatively few applications have so far been developed, and they have rarely been used to predict outcomes based on expert judgements (such as those that formed the basis of REF2014). The REF2014 exercise therefore provided a new possibility to test how useful prediction markets can be in predicting research evaluation exercises and assessments of scientific output.

We implemented a prediction market on the outcome of REF2014 for 33 chemistry departments in the UK. REF2014 publishes an overall quality profile for each department. This can be used to calculate a single number summary score (which REF2014 itself does not do), which we refer to as the REF2014 score (similar to a Grade Point Average): this simply weights 4* as 4, 3* as 3 and so on, and calculates the score as a weighted average.

## 2. Methods

We invited PhD students and researchers at all chemistry departments in the UK to participate in the prediction market. The prediction market was active for two weeks (17 November to 1 December 2014), with payments settled shortly after the outcome of the REF was released. The prediction market was implemented in collaboration with Consensus Point, a leading provider of prediction market research technology.

Before the market started, participants were asked to fill out a survey on their beliefs about the REF2014 score for each department (between 0 and 4, with one decimal), where we also asked them to rate how well they knew each institution (‘not at all’, ‘slightly’, ‘moderately’, ‘very well’ or ‘extremely well’, which we coded as a 1 to 5 variable). They were then given information on trading procedures as well as logins. Participants were given 10 000 points, the equivalent of £30, which they could use for betting on the different departments.

As REF2014 scores lie between 0 and 4, we created different intervals and let participants bet on these. The intervals were the following: score≤2, 2<score≤2.25, 2.25<score≤2.50, 2.50<score≤2.75, 2.75<score≤3.00, 3.00<score≤3.25, 3.25<score≤3.50, score>3.50. Participants traded (bought and sold) contracts that paid 100 points if the REF2014 score was in that interval, and 0 points otherwise. The type of contract we used in the prediction market allowed the price to be interpreted as the predicted probability (in per cent) of the outcome occurring. Although this price interpretation of the price has some caveats [8], it is simple and reasonably robust [9]. An estimated score is calculated based on the forecasted probability of each interval and its midpoint. For the outer intervals, we used 1.875 and 3.625 as midpoints (because using these gives the same distance between all midpoints). For all departments, the starting prices for all intervals were the same.

We used an automated market maker implementing a logarithmic market scoring rule that offers a buying price and a selling price at all times so that at all points there is a counterparty with which to trade [10]. This algorithm uses the net sales (*s*_1_…s_8_) the market maker has performed so far in the eight intervals for the score of a department to determine the prices for a (infinitesimally small) trade as *p*_*i*_=exp(−*s*_*i*_/*b*)/*Σ*_*j*=1…8_exp(−*s*_*j*_/*b*). Liquidity parameter *b* was set to 100.

For each interval for each department, participants could see the current price of that interval. When investing, and thus buying contracts in an interval for a specific department, participants selected this interval on the trading interface and entered the number of invested points. Each additional point invested in a position increased the price, and thus the predicted probability for this interval, while decreasing the price and the predicted probabilities for the other intervals. To sell, participants could decrease their position in an interval, which lowered the price for this interval.

On a separate part of the interface, participants could see a list of all departments where they had traded, and whether their position had increased or decreased in value. If the price of a contract had increased as the participant bought it (because other participants had bought the same contract), then the value of the position had increased. Conversely, if the price of a contract had decreased as the participant bought the contract (because other participants had sold this contract or bought contracts in other intervals), then the value had decreased. As participants had limited available points to trade with, they could choose to reduce the position in one contract to free points to invest in another contract.

## 3. Results

A total of 16 participants took part in the study, and had the opportunity to trade on 33 departments.^1^ Through the outcome of the prediction market, we obtained predicted scores for the 33 departments, shown in table 1. Furthermore, the survey data from the 16 participants were averaged to generate a survey-based prediction for each department, and weighted survey-based predictions were calculated by including the participant’s self-rated knowledge as weight.

**Table 1.** Predicted and actual REF2014 outcomes. (Overall score is calculated from outputs (65% weighting, 20% impact and 15% environment). GPA, grade point average.)

predicted (market)		overall (actual)		output (actual)		impact (actual)	
ranking	score	ranking	GPA	ranking	GPA	ranking	GPA
Cambridge	3.54	Cambridge	3.54	Liverpool	3.44	Durham	3.76
Imperial	3.26	Liverpool	3.50	Cambridge	3.42	Cambridge	3.66
Oxford	3.23	Oxford	3.43	Oxford	3.32	Liverpool	3.60
Manchester	3.18	Bristol	3.35	UEA	3.29	Manchester	3.50
Edinburgh/St Andrews^a^	3.15	Durham	3.31	Bristol	3.26	Leeds	3.50
UCL	3.10	UCL	3.30	Warwick	3.25	Bath	3.50
Bristol	3.09	Imperial	3.30	Queen Mary^c^	3.24	Cardiff	3.50
Durham	2.93	Warwick	3.30	Sheffield	3.21	UCL	3.49
Glasgow/Strathclyde^b^	2.82	Cardiff	3.29	Southampton	3.20	Oxford	3.47
Bath	2.88	UEA	3.27	Lancaster^c^	3.19	Imperial	3.47
Nottingham	2.86	Manchester	3.24	UCL	3.17	Bristol	3.43
Warwick	2.86	Southampton	3.23	Edinburgh/St Andrews^a^	3.17	Southampton	3.42
Leeds	2.86	Edinburgh/St Andrews^a^	3.23	Durham	3.17	York	3.40
Birmingham	2.85	Bath	3.23	Cardiff	3.17	Queen’s Belfast	3.40
Liverpool	2.85	Nottingham	3.22	Bath	3.16	Aberdeen	3.40
York	2.85	York	3.21	Imperial	3.15	UEA	3.40
Sheffield	2.84	Sheffield	3.19	York	3.13	Glasgow/ Strathclyde^b^	3.35
Southampton	2.80	Leeds	3.18	Nottingham	3.12	Warwick	3.30
Leicester	2.78	Glasgow/ Strathclyde^b^	3.15	Herriot-Watt	3.10	Newcastle	3.27
Herriot-Watt	2.75	Queen Mary^c^	3.07	Glasgow/ Strathclyde^b^	3.09	Leicester	3.23
Queen’s Belfast	2.75	Herriot-Watt	3.01	Birmingham	3.09	Sheffield	3.20
UEA	2.75	Birmingham	3.01	Manchester	3.08	Lancaster^c^	3.20
Cardiff	2.75	Lancaster^c^	2.98	Leeds	3.06	Edinburgh/St Andrews^a^	3.19
Sussex	2.74	Leicester	2.94	Reading	3.06	Nottingham	3.18
Hull	2.73	Aberdeen	2.94	Kent^c^	2.98	Hull	3.03
Aberdeen	2.71	Queen’s Belfast	2.93	Hull	2.96	Kent^c^	3.03
Loughborough	2.70	Newcastle	2.91	Leicester	2.89	Herriot-Watt	3.00
Bangor	2.70	Hull	2.91	Sussex	2.88	Loughborough	3.00
Newcastle	2.70	Kent^c^	2.88	Aberdeen	2.87	Queen Mary^c^	3.00
Reading	2.68	Reading	2.85	Queen’s Belfast	2.84	Birmingham	2.80
Huddersfield	2.59	Loughborough	2.70	Newcastle	2.84	Greenwich^c^	2.80
		Bangor	2.70	Huddersfield	2.76	Huddersfield	2.70
		Huddersfield	2.67	Bangor	2.73	Bangor	2.70
		Sussex	2.66	Loughborough	2.70	Reading	2.50
		Greenwich^c^	2.46	Greenwich^c^	2.53	Sussex	2.13

^a^Edinburgh and St Andrews submitted jointly.

^b^Glasgow and Stratchlyde submitted jointly.

^c^Greenwich, Kent, Lancaster and Queen Mary were not included in the prediction market.

The number of traders per department (i.e. the number of participants who bought or sold shares in a particular department) ranged from 0 to 15, with a median of 4 and a mean of 4.7, while the number of trades per department ranged from 0 to 36, with median of 5 and a mean of 6.9. This means that trading was relatively thin: typically trading did not occur in all of the eight intervals, and for more than half of the departments the market predictions are based on four or fewer participants, while for two departments (Heriott-Watt and Queen’s Belfast) there were no trades at all. We included all departments in our analysis because the lack of trading could be a consequence of traders not disagreeing with the initial market maker price, and because the market maker adjusts prices in all intervals change even if there are intervals that are not traded on.

Interestingly, the departments with the largest numbers of traders were the ones that subsequently received the best REF2014 scores. This might be because the best departments might also be the most visible ones (i.e. there is plenty of public information on the department), but also because these departments might be perceived to offer the most profitable bets because they are expected to deviate most from the initial pricing.

The scores predicted by the market were highly correlated with the prediction from the survey (coefficient=0.92, *p*=3.8×10^−13^) and weighted survey (coefficient=0.90, *p*=4.4×10^−12^), which is not unexpected given they both are based on the information held by the participants. However, average survey and weighted survey responses differed from the score predicted by the market in that they covered a different range (1.7 to 3.8 for the survey and weighted survey versus 2.6 to 3.5 for the market-based score), and had significantly different averages and variances (paired-samples *t*-test using survey averages and final market prices: *t*_30_=−3.2, *p*=0.003; *F*-test for comparing sample variances: *F*_30_=6.3, *p*=2.5×10^−6^).

The Pearson correlations between the market-based scores and the observed outcome (i.e. actual overall REF2014 score) was 0.71 (*p*=7.5×10^−6^), while the Pearson correlation coefficients between the survey-based predictions and observed outcome were somewhat larger: 0.80 (*p*=7.0×10^−8^) for simple averages, and 0.82 (*p*=2.3×10^−8^) for weighted averages. The Spearman rank correlations between predictions and outcomes were very similar for the three predictions: 0.83 (*p*=1.1×10^−8^) for the market-based prediction, 0.80 (*p*=8.5×10^−8^) for the survey, and 0.80 (*p*=6.1×10^−8^) for the weighted survey. The market systematically underestimated the mid-field, which explains why the Spearman correlation coefficients are stronger than the Pearson coefficients.

These correlations were very similar to the correlation between the 2008 Research Assessment Exercise (RAE2008, the predecessor of REF2014) score and the REF2014 score (Pearson: 0.84, *p*=2.8×10^−9^; Spearman: 0.79, *p*=1.2×10^−7^), although as with the survey predictions the RAE2008 scores fell in a different range to the REF2014 scores (because of a general increase in scores between RAE2008 and REF2014). The correlations between the market-based scores and the other REF2014 outcomes were weaker for both output (Pearson: 0.62, *p*=0.0002; Spearman: 0.67, *p*=3.3×10^−5^) and impact (Pearson: 0.50, *p*=0.004; Spearman: 0.66, *p*=5.9×10^−5^), which is unsurprising as market participants were asked to judge the overall REF2014 score.

**Figure 1.**
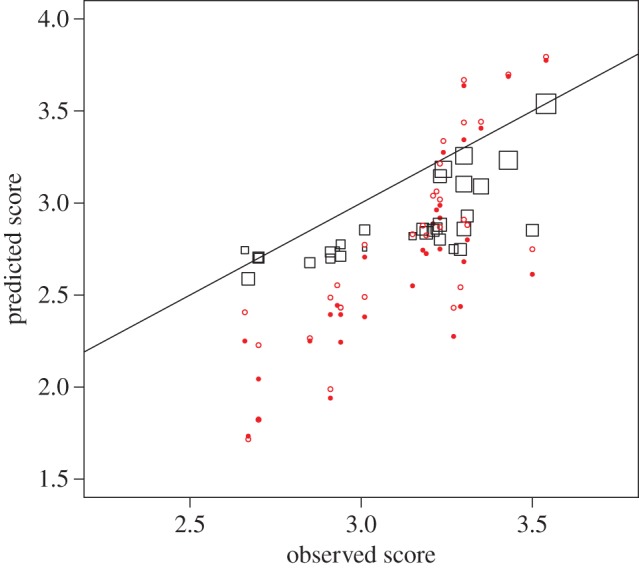
Comparison of performance of three methods of REF2014 prediction. The relationship between the predicted score (*y*-axis) and observed score (*x*-axis) is shown for overall REF2014 score, based on three prediction methods: survey (filled circle), weighted survey (open circle) and prediction market (square). Despite a higher correlation between survey-based predictions and observed outcomes, forecasting errors were lower for the market-based prediction, where the range of predictions matches the range of observed outcomes much better.

The predictions generated by the three methods are shown in figure 1. To compare the performance of the three methods, we calculated the forecasting error as the difference between predicted and observed score for each method. Welch two-sample *t*-tests comparing the market-based prediction and the survey measures showed that the forecasting error of the market-based score was significantly smaller than the survey-based forecasts (*t*_50.2_=−4.5, *p*=3.9×10^−5^ for simple averages, *t*_51.9_=−3.1, *p*=0.003 for weighted averages). Despite a higher correlation between survey-based predictions and observed outcomes, forecasting errors were lower for the market-based prediction, where the range of predictions matched the range of observed outcomes much better. In part, this is driven by the initial market maker pricing: the market particularly outperformed the survey for departments with a lower-than-average REF2014 score. For those departments, good initial pricing by the market maker in combination with thin trading resulted in good predictions.

We did not observe a significant impact of traded volume, number of traders or number of trades for a specific department on the forecasting error. This is because, for those markets with little trading activities, the initial market pricing resulted in a forecasted score close to the observed score. Thus, in this market segment, the initial values of the market contributed to the good market performance.

Institutions were in principle able to select only a proportion of their eligible staff in order to increase their REF2014 ranking. We used Higher Education Statistics Agency (HESA) contextual data for the REF2014 framework to estimate full-time equivalent (FTE) eligible staff. Note that REF2014 and HESA use different definitions, which can lead to deviations between the two records. We found that, for the chemistry departments included in our study, the fraction of REF2014 portfolio FTE’s to HESA eligible FTE’s (REF : HESA ratio) ranged from 47% to 108%. The highest value was obtained for Cambridge, for which HESA states that there are some REF2014-eligible staff that are not included in HESA figures. The median and mean were 91% and 88%, respectively.

To test whether those institutions that were more selective in their REF2014 submission obtain better-than-expected outcomes than institutions that were less selective, we calculated the difference between outcome and expectation and related it to the REF : HESA ratio. We observed strong correlations of the REF : HESA ratio when expectation was derived from survey averages and weighted survey averages (coefficient=−0.68, *p*=3.0×10^−5^; coefficient=−0.73, *p*=3.3×10^−6^), but no evidence of a strong correlation between the REF2014 : HESA ratio when expectation was derived from final market prices (coefficient=−0.21, *p*=0.25). Therefore, at least the survey averages suggest that selective institutions had unexpectedly high outcomes.

## 4. Discussion

Our results suggest that prediction markets can predict the outcome of research evaluation exercises based on expert judgements such as REF2014. In principle, they could be a promising tool for planning purposes for the leadership of HEI’s, could help replacing costly and time-consuming mock exercises, and perhaps could even help reducing the efforts associated with actual REF evaluation itself. Conceivably, given the speed with which prediction markets can be deployed and updated, they could be run at regular intervals by departments of institutions to assess whether strategic decisions intended to improve research quality are having the desired impact. Their utility, therefore, potentially extends beyond simply predicting the results of research evaluations.

We did observe a few mismatches between the market prediction and the REF2014 outcome. For example, Liverpool and Cardiff did relatively well in the REF2014 outcome, whereas Imperial, Manchester and Edinburgh did rather poorly. This may reflect strategic decisions made by these institutions—for example, trading-off research quality by returning a large number of portfolios, to maximize research income at the expense of ranking (because the financial award is a function of both the REF2014 score and the size of each individual submission—the number of FTE staff). Indeed, we found some evidence of this on the basis of the survey data, which suggested that selective institutions had unexpectedly high outcomes. Alternatively, it might be possible to reduce the prediction error by improving the procedures for the market and increasing the number of participants. For example, the difference in the range of distributions of the survey score and the market-based score may be due to having a binary outcome in one case and a continuous in the other, but may also be an effect of liquidity—in the REF2014 market, because of a relatively low number of participants, we had limited liquidity, which may have limited the participants’ ability to drive forecasts further apart.

There are some limitations to our approach that should be considered. First, we asked participants in the market to predict the average REF2014 score. However, ultimately the REF2014 exercise will be used to allocate funding, and this is likely to be the outcome that HEIs are most strongly motivated by. As we have seen, the funding allocation is decided as a function of both the REF2014 score and the size of each individual submission. Nevertheless, if the market is fully efficient it should be able to incorporate all information related to the REF2014 score, including how institutions may strategize to obtain more funding (for example, by increasing the size of their submission). Therefore, a market could instead ask participants to directly predict the funding allocation. Second, the number of participants in the market was limited, possibly because the concept of prediction markets is unfamiliar to many scientists outside of economics, and the invitation to participate was unsolicited. With only 16 participants trading on 33 departments, each of which included eight interval markets, the level of trading was relatively thin. A larger number of participants across more institutions should improve the accuracy of the prediction market further, and would diminish the impact of the initial market maker pricing on the final predictions. Third, we only assessed one UoA (chemistry). This was for pragmatic reasons—we did not have the resources to run multiple markets across several UoAs. We also needed to select a UoA that mapped closely onto traditional departmental structures (in order to accurately invite relevant individuals to participate in the market). We chose chemistry principally because this was one case where a traditional university structure (i.e. a single department) mapped on to a single UoA. We explored the possibility of including another UoA (philosophy) but were unable to recruit participants into this market. We, therefore, cannot say with certainty whether our results would translate to other UoAs, although we have no reason to think they would not. Nevertheless, it is worth noting that Mryglod *et al.* [11] report that departmental *h*-indices predicted REF outcomes to varying degrees across the different UoAs they considered. Fourth, we used departments as the unit of observation in our analyses, but ratings of departments were obtained from the same participants, which raises the question of whether this introduces a problem of non-independence. However, in our opinion, it is unlikely that this would considerably influence our results. Relatedly, it is also worth noting that institutions and departments themselves may not be statistically independent—there may be geographical dependencies, for example, and indeed we see evidence of this in institutions making joint submissions to REF2014. Unfortunately we lack the data to investigate potential dependencies. Nevertheless, we again think it is unlikely that this would considerably influence our results, although this may be a subject for future study.

There has been interest in whether a metric-based approach could have achieved much the same as REF2014 at a fraction of the cost and effort. One approach that has been suggested is to rank departments on the basis of their departmental *h*-index [12], to capture both the productivity and citation impact of individual departments. There are a number of metrics (prediction market outcomes, departmental *h*-indices, departmental size and more general HEI rankings such as those published by Times Higher Education) that correlate more or less strongly with REF2014 scores and (perhaps more importantly) the amount of funding ultimately allocated to individual departments [1]. However, a limitation of metric-based approaches is that individual departments often do not map directly onto the UoAs included in REF2014. It has also been reported that departmental *h*-indices only weakly predicted actual REF2014 outcome in biology, chemistry, physics and sociology [11]. Crucially, there is also considerable skepticism among researchers and institutions about the use of metrics in research assessment [13].

One possibility for future REF exercises might be to conduct a full assessment on a random subset of departments, so that there is an incentive for participants in the market to predict truthfully. This information could then be used, together with the results of the full assessment, to extrapolate the results to those departments not assessed. However, even if a strongly correlated predictor of the evaluation is identified, there can be substantial differences in the finances awarded based on the predictor and the actual evaluation. Although this is difficult to accurately estimate, not least because the formula used to allocate funding can change between assessment exercises, any discrepancy is likely to be unpalatable to institutions awarded funding on the basis of a predicted score rather than an actual score. Indeed, we observed notable discrepancies in the ranking of individual institutions when comparing the rankings according to the market and the actual rankings. Similarly, while the size of the submission (i.e. the number of FTE staff) correlates strongly with the financial amount awarded, it is likely that awarding funds solely on the basis of size, with no reference to quality, would also be unacceptable. We, therefore, do not suggest that prediction markets are a suitable replacement for research evaluation exercises. Principally, prediction markets need to operate in relation to a subsequent real outcome.

Nevertheless, given the substantial expense associated with REF2014 (estimated at almost £60 million [1], although other estimates place the total cost at closer to £250 million [14]), there is clearly a need to explore whether simpler, cheaper methods of assessing research quality exist that could form part of any future assessment exercise. Further research is required to establish how incentives to ‘game’ such a market could be counteracted. In particular, as any system can be gamed, one critical question is whether the consequences of any gaming under a cheaper, less onerous REF process would be any worse than under the current system. If not, then the benefits of any savings associated with a lighter-touch REF process may be considerable and less likely to be offset by any unintended consequences.

## 5. Conclusion

Evaluations such as REF2014 are interesting in terms of information disclosure, because individuals with best knowledge of their performance also have the strongest incentives to signal a strong performance. This seems to make costly external assessment inevitable. Creating carefully designed mechanisms for information revelation in such a situation might be challenging, but not impossible. Prediction markets are a first step in this direction.

## Footnotes

1 These 33 departments did not include all departments that were later in the 2014 REF. Institutions that are not included in the market are excluded from the analysis.

## Data accessibility

The data underlying the analyses reported in this study can be obtained from the University of Bristol Research Data Repository (http://data.bris.ac.uk/data/), doi:10.5523/bris.1s8bmxopxry716vkmluw1aii5.
